# Seed Sown by the Wayside

**Published:** 1885-12

**Authors:** E. M. Walton

**Affiliations:** Georgetown, Texas


### SEED SOWN BY THE WAY-SIDE.

We met Dr. Walton in Austin recently, and we asked him if they had a medical society in Williamson county. He said they had not. We urged him, on his return home, to exhort the profession to turn out and get up an organization of the regular physicians in the county. As an outcome of that conversation, we have received the following most gratifying intelligence. Of course we will go. The mission of the JOURNAL is thus being fulfilled, and we are proud of our work in that line.

GEORGETOWN, TEXAS, Dec. 5, 1885.

DR. F. E. DANIEL, Austin, Texas :

DEAR DOCTOR :—I am glad to write you that the physicians of our town have at last awaked from their "Rip Van Winkle" sleep; and, to-day, took the first steps toward reorganizing the County Medical Society. We met and appointed a committee, who are to draft a circular setting forth the necessity for reorganization, and calling upon all *regular* practitioners in the county to meet with us in Georgetown, on the first Thursday in January.

When I was in Austin, recently, it was my pleasure to meet you, and you were kind enough to offer to meet with us and give us the benefit of your counsel and your experience in organizing medical societies. I mentioned this to the doctors, and they requested me to write and ask you if you will come up and assist us; and if you will also favor us with an address, such as you may think suited to the occasion; something that will arouse our doctors to a sense of their duties in the protection of themselves and brethren against the quacks and impostors who are daily creeping in amongst us; and, greater than this, to a sense of the need for mutual interchange of ideas and experience for the advancement of medical science.

Now, Doctor, if you can so arrange your affairs as to be with us, we shall not only be very greatly pleased, but we will also rest under many obligations to you.

Please let us hear from you just as soon as possible, because if you can be present with us and favor us with the address, we wish to so state in our circular letter to physicians.

Yours, very truly,

E. M. WALTON.

